# CCT: continuous care trial - a randomized controlled trial of the provision of continuous care during labor by maternity care assistants in the Netherlands

**DOI:** 10.1186/s12884-020-03336-6

**Published:** 2020-11-25

**Authors:** Adrie Lettink, Karina Chaibekava, Luc Smits, Josje Langenveld, Rafli van de Laar, Babette Peeters, Marie-Louise Verstappen, Carmen Dirksen, Marianne Nieuwenhuijze, Hubertina Scheepers

**Affiliations:** 1 Department of Obstetrics & Gynecology, Maastricht University Medical Center (MUMC+), Peter Debyelaan 25, 6229 HX Maastricht, The Netherlands; 2 Department of Obstetrics & Gynecology, Maastricht University Medical Center (MUMC+), Maastricht University, Peter Debyelaan 25, 6229 HX Maastricht, The Netherlands; 3 Faculty of Health, Medicine and Life Sciences, Department of Epidemiology, Maastricht University, Peter Debyeplein 1, 6229 HA Maastricht, The Netherlands; 4 Department of Obstetrics & Gynecology, Zuyderland Medical Center, Heerlen, The Netherlands; 5 Department of Obstetrics & Gynecology Viecuri Medical Center, Venlo, The Netherlands; 6 Department of Maternity Care Assistants, Cicogna Kraamzorg, Oude Rijksweg 32, 6271 AA Gulpen, The Netherlands; 7 Department of Maternity Care Assistants, Geboortezorg Limburg, Minckelersstraat 2, 6372 PP Landgraaf, The Netherlands; 8 Health Technology Assessment, Maastricht University, Peter Debyeplein 1, 6229 HA Maastricht, The Netherlands; 9 Research Center for Midwifery Science, Zuyd University, Universiteitssingel 60, 6229 ER Maastricht, The Netherlands; 10 Gynecologist, Department of Obstetrics & Gynecology, Maastricht University Medical Center (MUMC+), Maastricht University, Peter Debyelaan 25, 6229 HX Maastricht, The Netherlands; 11 School for Oncology and Developmental Biology (GROW), Maastricht University, Peter Debyelaan 25, 6229 HX Maastricht, The Netherlands

**Keywords:** Continuous support, Epidural analgesia, Labor, Childbirth, Experience, Healthcare costs

## Abstract

**Background:**

In 2009, the Steering Committee for Pregnancy and Childbirth in the Netherlands recommended the implementation of continuous care during labor in order to improve perinatal outcomes. However, in current care, routine maternity caregivers are unable to provide this type of care, resulting in an implementation rate of less than 30%. Maternity care assistants (MCAs), who already play a nursing role in low risk births in the second stage of labor and in homecare during the postnatal period, might be able to fill this gap. In this study, we aim to explore the (cost) effectiveness of adding MCAs to routine first- and second-line maternity care, with the idea that these MCAs would offer continuous care to women during labor.

**Methods:**

A randomized controlled trial (RCT) will be performed comparing continuous care (CC) with care-as-usual (CAU). All women intending to have a vaginal birth, who have an understanding of the Dutch language and are > 18 years of age, will be eligible for inclusion. The intervention consists of the provision of continuous care by a trained MCA from the moment the supervising maternity caregiver establishes that labor has started. The primary outcome will be use of epidural analgesia (EA). Our secondary outcomes will be referrals from primary care to secondary care, caesarean delivery, instrumental delivery, adverse outcomes associated with epidural (fever, augmentation of labor, prolonged labor, postpartum hemorrhage, duration of postpartum stay in hospital for mother and/or newborn), women’s satisfaction with the birth experience, cost-effectiveness, and a budget impact analysis. Cost effectiveness will be calculated by QALY per prevented EA based on the utility index from the EQ-5D and the usage of healthcare services. A standardized sensitivity analysis will be carried out to quantify the outcome in addition to a budget impact analysis. In order to show a reduction from 25 to 17% in the primary outcome (alpha 0.05 and bèta 0.20), taking into account an extra 10% sample size for multi-level analysis and an attrition rate of 10%, 2 × 496 women will be needed (*n* = 992).

**Discussion:**

We expect that adding MCAs to the routine maternity care team will result in a decrease in the use of epidural analgesia and subsequent costs *without* a reduction in patient satisfaction. It will therefore be a cost-effective intervention.

**Trial registration:**

Trial Registration: Netherlands Trial Register, NL8065. Registered 3 October 2019 - Retrospectively registered.

## Background

Childbirth is a life-changing event for all women, resulting in various emotions. In modern society, birth often takes place in a clinical environment, which may increase feelings of loss of control and anxiety [1]. Studies show that 20% of women in high-income countries fear childbirth. These women are more likely to request an epidural or even a caesarean section [2]. Anxiety in pregnancy is associated with more interventions during birth. Randomized controlled trials have shown that women who are provided with continuous support during labor and birth need fewer medical interventions, have improved neonatal outcomes, and report a more positive childbirth experience [1]. Women benefit from informational, emotional, physical and practical support during labor [3]. A member of staff, a doula, or a lay-person from the woman’s own network may offer continuous support in the form of improved communication, the provision of coping or massage techniques, assistance with finding the right position, and help with decision-making. This kind of support has been shown to improve both women’s satisfaction with birth experience and childbirth outcomes [1, 3–7]. Subgroup analysis in a Cochrane systematic review suggested that support was most effective when provided by someone in a (trained) doula role [1]. Both the World Health Organization and the Steering Committee for Pregnancy and Childbirth in the Netherlands have recommended the provision of continuous support during labor [1, 8], but the actual implementation of this support remains a challenge. In a pilot study conducted by the Royal Dutch Organization of Midwives, 101 of 125 participating women (80%) indicated that they wanted to receive continuous care during labor, and a reduction in both transfer to secondary care and request for pain medication was observed when this care was provided [9, 10].

In the Netherlands, midwives provide pregnancy, birth and postnatal care to healthy, low-risk women. These women can choose to give birth at home or in a hospital. Based on national guidelines [11], a risk assessment is performed for each pregnant woman during pregnancy and delivery. This risk assessment can result in transfer of care between midwife and obstetrician at any time during pregnancy or delivery. Maternity care assistants (MCAs) assist the midwife during the last part of the first stage and in the second stage of birth. For both low- and high-risk women, midwives and MCAs provide home care following hospital discharge, for the first week of the postnatal period.

Currently, the resources are not available to provide continuous care to all women during labor in routine midwifery- or obstetric-led care. In fact, continuous care is provided in less than 30% of all cases [12]. At the same time, nationwide, there has been a steep rise in the use of epidural analgesia (EA) amongst primiparous women, from 17% in 2006 to 43% in 2018. In our region, more than half of all women delivering their first baby uses EA. [12] EA is associated with transfer of care, maternal fever, an increased risk of an instrumental delivery, and admittance of the baby due to antibiotic treatment [13]. These outcomes also result in a considerable rise in costs. We hypothesize that adding MCAs to standard maternity care for the provision of continuous support during delivery will be a cost-effective intervention. We predict that any extra costs associated with providing this care will be outweighed by the savings associated with the reduction in EA use. Finally, we predict that the intervention will not decrease patient satisfaction or lead to an increase in complications.

## Methods

### Study design

This Continuous of Care Trial (CCT) will be a two-armed, multi-center randomized controlled trial in midwifery-led and obstetric-led care facilities in the southeastern region of the Netherlands. It will compare the effects of continuous care (CC) provided by a trained maternity care assistant during labor (intervention group, CC group) to care-as-usual (control group, CAU group). We define continuous care as care that is given during the entire first stage of labor by a trained maternity care assistant (MCA), who can offer emotional, informational, physical and practical support. While having no direct medical responsibilities (due to level of training and experience) the MCA is trained in signaling Obstetric emergencies and therefore able to contact the responsible care provider when necessary. MCAs will be asked to be present at the birth from the moment the responsible maternity care provider states that labor has started.

### Study setting and population

All primiparous and multiparous women planning to have a vaginal birth in the southeastern region of The Netherlands will be eligible to participate in this study, including women planning a home or hospital birth in midwifery-led and obstetric-led care. Exclusion criteria will include being under the age of 18, being unable to speak, read or write the Dutch language, and women with a planned cesarean delivery.

### Study outcomes

Our main outcome measure will be the use of epidural analgesia (EA). Secondary outcomes will be (1) referrals from primary care to secondary care, (2) caesarean delivery, (3) instrumental delivery, (4) adverse outcomes associated with epidural (fever, augmentation of labor, prolonged labor, postpartum hemorrhage, duration of postpartum stay in hospital for mother and/or newborn), (5) cost-effectiveness, (6) budget impact analysis and (7) women’s satisfaction with the birth experience.

### Sample size calculation

We calculated that, in order to be able to detect a reduction in use of EA from 25 to 17% (with an alpha 0.05 and beta 0.20), taking into account the use of multilevel analysis (10% higher sample size) and an attrition of 10% (90% complete records), a sample size of 2 × 496 participants will be necessary (*n* = 992).

### Recruitment

The intervention will be provided by the two largest maternity care agencies in the southeastern region of Limburg. The recruitment will be carried out by intake-staff of the maternity care agencies, who have been trained by the principal investigator. Each pregnant woman registering for postnatal care at one of these agencies (usually in the first trimester of pregnancy) will be informed about the study by telephone or e-mail and will then receive further information by telephone or e-mail. At the standard home visit around 34 weeks of gestation, the intake-staff will answer any remaining questions about the study. If the woman is willing to participate, an informed consent form will be signed. To optimize recruitment, all midwifery practices and the participating hospitals will be informed about the study and asked to inform the women in their care about the study.

### Randomization

Once informed consent has been given, the participant will be asked to fill out some baseline questionnaires. Subsequently, participants will be randomly assigned (using sealed, blank envelopes) to either the intervention or the care-as-usual group. A list based on block randomization with a random block size, using https://www.randomizer.org, will be used for the order in which to fill the envelopes. Randomization will be stratified for different maternity care agencies to prevent the occurrence of any potentially confounding variables based on the location of the two agencies. Stratification will also be done for parity; this is an important determinant of use of EA with a two to three-fold higher uptake in primiparous women compared to multiparous women [12, 14].

### Intervention

If a woman has been randomly assigned to continuous care, she (or her partner) will call the MCA for support at the onset of labor. Since this can be a subjective interpretation, we define the onset of labor as the moment the midwife or doctor confirms that labor has started. In the case of induction of labor, we decided that the MCA will be called for continuous support at the point in time when the membranes are artificially ruptured. On average, the MCA needs approximately 45 min to get to the destination. The MCA will provide care that is tailored to the needs of the woman in labor, such as helping with relaxation, offering massage or breathing exercises, or being present and giving verbal reinforcement. The duration of CC is defined as the moment the MCA arrives until the start of active pushing in the second stage of labor.

### Data collection

Data will be collected at three moments in time (Table 1). Baseline demographic and obstetric characteristics will be collected before randomization. At this point, the questionnaire also includes:

- The EQ5D, a standardized validated instrument measuring five health dimensions (mobility, self-care, daily activities, pain and discomfort, and anxiety and depression) that allows us to asses health in individuals or populations [15, 16];
- The Cambridge Worry Scale (CWS), a validated instrument used to measure the extent and content of worries in specific situations (socio-medical, own health, socio-economic and relational) [17];
- The State-Trait Anxiety Inventory (STAI) questionnaire, a validated instrument that measures two types of anxiety: state and trait [18, 19].

**Table 1:**
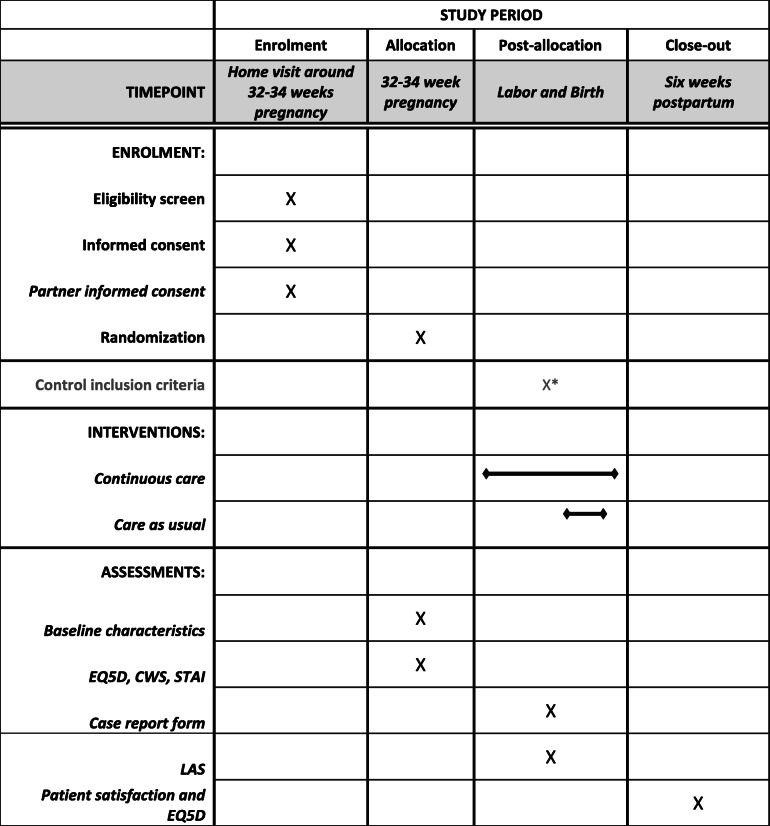
Timeline and Data Collection. EQ. 5 D Standardized validated instrument measuring five health dimensions. STAI State-Trait Anxiety Inventory

*Control of in- and exclusion criteria (i.e. reason for removal from study: elective caesarean because of breach position).

*EQ5D* Standardized validated instrument measuring five health dimensions; *STAI* State-Trait Anxiety Inventory; *CWS* Cambridge Worry Scale; *LAS* Labor Agentry Scale; *CLIK-Q* Questionnaire to reflect on patient satisfaction (maternity empowerment/experience)

In the first week after birth (preferably the same day of the birth), the MCA will fill-out a case report form together with the midwife or doctor, based on primary and secondary outcome measures. Additionally, the woman will fill out the Labor Agentry Scale (LAS) questionnaire, which scores experience of personal control during labor. In field studies, the LAS showed an inverse relationship between anxiety and control [20].

Six weeks after birth, the participants will fill out the final questionnaire, the CLiK questionnaire, used within Dutch maternity care agencies to reflect on patient satisfaction. It is based on the validated Maternity Empowerment Questionnaire. Questions are about maternity empowerment, the EQ5D and women’s experiences with maternity care assistance. We will perform random checks of the completed case reports and we will extract data from the main source if there are inconsistencies in completed case report forms.

### Data management

Data will be collected on paper and will be temporarily stored in a safe location at the University of Maastricht. Subsequently, after digitalizing, the data will be stored for 15 years in the research department of the Maastricht University Medical Center. We will use MACRO to digitalize all data.

### Statistical analysis

Baseline characteristics include age in years, ethnic background, gravidity and parity, gestational age at birth, mode of birth in this pregnancy, and if applicable, mode of previous birth and experience of previous birth(s). The primary outcome EA, and its association with continuous care versus care-as-usual, will be analyzed using multilevel logistic regression analysis.

The secondary outcomes are listed in Table 2, and will be addressed using multilevel linear regression analyses (for continuous variables), multilevel logistic regression (for binary variable variables) or multilevel multinomial logistic regression analysis (for categorical outcomes with more than two categories). If important baseline characteristics will differ greatly between the study arms, we will include them in the analysis. Missing data will be imputed by use of stochastic regression imputation, with predictive mean matching as the imputation model. We will perform both an intention to treat analysis and per protocol analysis. Cost-effectiveness will be calculated for the six-week period following childbirth and incremental cost effectiveness ratios will be calculated in QALY per prevented EA based on utility index from the EQ5D and usage of healthcare. Standardized sensitivity analysis will be carried out to quantify the outcome. A budget impact analysis will be performed using ISPOR guidelines, which take into account the financial consequences of the implementation of maternity care assistants as providers of continuous support during labor.

**Table 2:** Secondary Outcome Measures

**Maternal outcome**	
Mode of delivery	
3th or 4th degree perineal tear	
Use of pain medication other than Epidural Analgesia	
> 1000 cc blood loss	
Blood transfusion	
Fever during labor	
Use of antibiotics	
Proven infection	
Uterine rupture	
Intensive Care (IC) admittance	
Maternal death	
**Neonatal outcome**	
Need for admittance to hospital	
Neonatal Intensive Care Unit (NICU) admittance	
Apgar below 7	
Neonatal death	
**Health care use**	
Transfer of care from midwifery-led to obstetric-led care	
Duration of	
- continuous care	
- home care	
- hospital care	
- Neonatal Intensive Care Unit	
- Intensive Care	
**Questionnaires**	
EQ 5D^a^State-Trait Anxiety Inventory (STAI)	
Cambridge Worry Scale (CWS)	
Labor Agentry Scale (LAS)	
CLIK-Q^b^	

^a^ Standardized validated instrument measuring five health dimensions

^b^ Questionnaires to reflect on patient satisfaction (maternity empowerment and experience)

One hour of care provided by an MCA costs approximately 45 euros, and we expect extra costs of approximately 225 euros per woman for the provision of continuous care. The costs for one EA, when taking complications into consideration, is approximately 2200 euros. We would therefore expect the use of continuous care to be cost effective with just a 10% reduction in EA use.

## Discussion

Our study will determine whether the implementation of continuous care during labor will result in a lower use of EA - leading to fewer complications and reducing costs - without lowering women’s level of satisfaction with the birth experience.

## Data Availability

There is no online database. Once completed there will be a digital database with no trace to confidential data at University of Maastricht. If interested or questions on the data please contact corresponding author: Adrie Lettink.

## Abbreviations

EA: Epidural analgesia
CCT: Continuous Care Trial
CC: Continuous Care group (intervention)
CAU: Care as Usual group (control)
MCA: Maternity Care Assistant
SES: Social Economic Status

## References

[1] Bohren MA, Hofmeyr GJ, Sakala C, Fukuzawa RK, Cuthbert A (2017). Continuous support for women during childbirth. Cochrane Database Syst Rev.

[2] Nieminen K, Malmquist A, Wijma B, Ryding EL, Andersson G, Wijma K (2015). Nulliparous pregnant women's narratives of imminent childbirth before and after internet-based cognitive behavioural therapy for severe fear of childbirth: a qualitative study. BJOG.

[3] Kabakian-Khasholian T, El-Nemer A, Bashour H (2015). Perceptions about labor companionship at public teaching hospitals in three Arab countries. Int J Gynaecol Obstet.

[4] Kobayashi S, Hanada N, Matsuzaki M, Takehara K, Ota E, Sasaki H, Nagata C, Mori R (2017). Assessment and support during early labor for improving birth outcomes. Cochrane Database Syst Rev.

[5] Counselling for Maternal and Newborn Health Care: A Handbook for Building Skills. Geneva: World Health Organization; 2013. 10, SUPPORT DURING LABOUR AND CHILDBIRTH. Available from: https://www.ncbi.nlm.nih.gov/books/NBK304186/ accessed on Oct 2019. 26158182

[6] Backes Kozhimannil K, Hardeman R, Attanasio L, Blauer-Peterson C, O’Brien M. Doula Care, Birth Outcomes, and Costs Among Medicaid Beneficiaries. Am J Public Health. 103:e113–21. 10.2105/AJPH.2012.301201 PMC3617571 23409910

[7] Lunda P (2018). Women’s experiences of continuous support during childbirth: a meta-synthesis. BMC Pregnancy and Childbirth..

[8] Stuurgroep zwangerschap en Geboorte, een goede start, Utrecht December 2009 [ Steering comitee pregnancy and birth. A good start. Utrecht December 2009 ]. https://www.nvog.nl/wp-content/uploads/2018/02/Advies-Stuurgroep-zwangerschap-en-geboorte-1.0-01-01-2009.pdf Accessed on Sept 2018.

[9] Weide M, Bruinsma A (2013). Pilot studie continue begeleiding KNOV; febr 2013 [ A pilot study on continuous support during labor. KNOV].

[10] van Driel W, Hakkenberg R. Handreiking "continue begeleiding tijdens de bevalling bij vrouwen met een medische indicatie” KNOV 2011–2012 [ Continuous care during labour in obstetricianledcare in the Netherlands] https://www.knov.nl/vakkennis-en-wetenschap/tekstpagina/537-2/continue-begeleiding-tijdens-de-baring/hoofdstuk/1279/pilots-en-handreikingen/ Accessed on Sept 2018.

[11] Verloskundig Vademecum 2003. Eindrapport van de Commissie Verloskunde van het College voorzorgverzekeringen. Diemen, The Netherlands: College voor Zorgverzekeringen; 2003 [ Obstetric Vademecum 2003. Final report of the Maternity Care Committee of the College of health insurance companies ]. http://geboortebeweging.nl/wpcontent/uploads/2015/10/verloskundigvademecum2003.pdf Accessed on Sept 2018.

[12] Perined. Perinatale Zorg in Nederland 2016. [ Perined perinatal care in the Netherlands ] https://assets.perined.nl/docs/7935f9c6-eaac-4f59-a150-307ae04efa27.pdf Accessed on Sept 2018.

[13] Wassen M, Zuijlen J, Roumen F, Smits L, Marcus M, Nijhuis J (2011). Early versus late epidural analgesia and risk of instrumental delivery in nulliparous women: a systematic review. BJOG.

[14] Wassen M (2014). Epidural analgesia and operative delivery: a ten-year population-based cohort study in The Netherlands. Eur J Obstet Gynecol Reprod Biol.

[15] Salén BA, Spangfort EV, Nygren AL, Nordemar R (1994). The disability rating index: an instrument for theassessment of disability in clinical settings. J Clin Epidemiol.

[16] Hurst NP, Kind, P, Ruta D, Hunter M, Stubbings A. Measuring health-related quality of life in rheumatoid arthritis: validity, responsiveness and reliability of EuroQol (EQ-5D). Br Rheumatol1997;36(5):551–559. 10.1093/rheumatology/36.5.551 9189057

[17] Green JM, Kafetsios K, Statham HE, Snowdon CM (2003). Factor structure, validity and reliability of the Cambridge worry scale in a pregnant population. J Health Psychol.

[18] Spielberger CD (1989). State-trait anxiety inventory: bibliography.

[19] Spielberger CD, Gorsuch RL, Lushene R, Vagg PR, Jacobs GA (1983). Manual for the state-trait anxiety inventory.

[20] Ellen D. Hodnett, Daryl A. Simmons-Tropea, The labour agentry scale: psychometric properties of an instrument measuring control during childbirth, First published: October 1987 https://doi-org.ezproxy.ub.unimaas.nl/10.1002/nur.4770100503. 10.1002/nur.4770100503 3671777

